# Robust Vector BOTDA Signal Processing with Probabilistic Machine Learning

**DOI:** 10.3390/s23136064

**Published:** 2023-06-30

**Authors:** Abhishek Venketeswaran, Nageswara Lalam, Ping Lu, Sandeep R. Bukka, Michael P. Buric, Ruishu Wright

**Affiliations:** 1 National Energy Technology Laboratory, 626 Cochrans Mill Road, Pittsburgh, PA 15236, USA; 2 NETL Research Support Contractor, 626 Cochrans Mill Road, Pittsburgh, PA 15236, USA; 3 National Energy Technology Laboratory, 3610 Collins Ferry Road, Morgantown, WV 26505, USA

**Keywords:** optical fiber sensors, data analytics, deep neural networks, distributed fiber sensors, sensor data

## Abstract

This paper presents a novel probabilistic machine learning (PML) framework to estimate the Brillouin frequency shift (BFS) from both Brillouin gain and phase spectra of a vector Brillouin optical time-domain analysis (VBOTDA). The PML framework is used to predict the Brillouin frequency shift (BFS) along the fiber and to assess its predictive uncertainty. We compare the predictions obtained from the proposed PML model with a conventional curve fitting method and evaluate the BFS uncertainty and data processing time for both methods. The proposed method is demonstrated using two BOTDA systems: (i) a BOTDA system with a 10 km sensing fiber and (ii) a vector BOTDA with a 25 km sensing fiber. The PML framework provides a pathway to enhance the VBOTDA system performance.

## 1. Introduction

Brillouin-based distributed fiber-optic sensors have gained tremendous attention over the past two decades due to their capability of measuring both strain and temperature over tens of kilometers. A wide range of structural health monitoring applications on large civil infrastructure (oil and gas pipelines, bridges, and railways), power grids, and border security have been demonstrated [1,2]. Brillouin optical time-domain analysis (BOTDA) is based on stimulated Brillouin scattering, where pump pulses and probe continuous waves (CWs) injected into a fiber from both ends excite acoustic waves to facilitate power coupling between the two counter-propagating optical waves. The pulsed pump waves generate a gain or loss for the CW probe waves with downshifted or upshifted frequencies. A Brillouin gain/loss spectrum (BGS/BLS) can be obtained over the sensing fiber distance by sweeping frequencies around the Brillouin frequency shift (BFS), which depends on the strain and temperature of the fiber. Several vector BOTDA (VBOTDA) system configurations have been proposed to extract both the BGS and Brillouin phase spectrum (BPS) simultaneously. The BPS is independent of Brillouin gain and is unaffected by nonlocal effects. BPS exhibits low noise and is therefore a good target for deriving sensor information [3,4,5,6]. For instance, an IQ demodulation algorithm was proposed with heterodyne detection and measured both the BGS and BPS [7]. A double-frequency phase modulation method was proposed that provides measurements of both BGS and BPS [8]. Recently, VBOTDA was proposed by using a vector network analyzer to acquire BGS and BPS [9,10].

The incident pump pulses at a frequency $\nu_{0}$ with a certain pulse width counter-propagates to a CW probe wave at a frequency $\nu_{0}-\nu_{B}$. The input pulses generate a backscattered Brillouin signal, which is downshifted to the pump signal by the BFS $\nu_{B}$ (around 10–11 GHz for silica single-mode fibers). The probe experiences Brillouin amplification and Brillouin phase shift when the difference between the input pump and probe wave frequency is around the BFS. A suitable signal processing technique must be employed to evaluate the BFS from the measured BGS/BPS over the fiber length. Several signal processing techniques have been proposed in the literature to estimate the BFS from BGS and/or BPS measurements. These techniques can be categorized as either (i) curve fitting (CF) or (ii) machine learning (ML)-based approaches. The latter approach is increasingly being favored for long-distance BOTDA systems for its capability to process data in real time. Several studies in the literature have reported that the ML-based models have provided more accurate BFS predictions than their CF counterparts in scenarios such as (i) low signal-to-noise ratio and (ii) coarsely resolved BGS/BPS measurements [11,12]. ML techniques can indeed be combined with Brillouin-based distributed fiber sensors to enhance their performance and to enable advanced signal processing and data analysis. These advancements open up opportunities for a wide range of intelligent monitoring applications, including structural health monitoring, environmental monitoring, and smart infrastructure management. In the literature, several efforts are being made to address these challenges and to develop robust and efficient ML algorithms that can accelerate the signal processing speed and handle the complexity and volume of data generated by distributed fiber sensors.

BOTDA systems are often deployed in harsh environments over long distances and are prone to several environmental and systemic factors that can increase sensor noise and can degrade the performance of the sensing system. Several factors reduce the SNR of a BOTDA system, such as the strength of the backscattered Brillouin signal (typically at nW), silica fiber double path loss (typically 0.4 dB/kmat 1550 nm), noise sources, and other nonlinear effects that limit the input power of pumps and probes [13]. Polarization noise, relative intensity noise (RIN), amplified spontaneous emission (ASE) noise, and thermal and shot noise are the major noise sources that degrade the SNR [14].

A data processing algorithm based on radial basis function neural networks (RBFNN) was proposed in [15], for BOTDR sensing. The RBFNN algorithm was shown to accelerate data processing compared with the Levenberg–Marquardt nonlinear least-squares algorithm. A recurrent neural network-based data processing framework was demonstrated [16] on data collected using a commercial Brillouin-based distributed temperature sensing system (AP Sensing, N4385B). This approach utilized an autoregressive input layer often found in time series analysis literature as autoregressive models. A ML-based algorithm using support vector machines [17] was used to extract BFS from both gain and phase data collected from a BOTDA sensing system. The proposed algorithm improved the data processing speed by 100 times compared with the conventional nonlinear least squares data processing technique. Deep neural networks (DNNs) with autoencoder architectures were introduced to extract BFS from BOTDA data [11,18]. A BOTDA sensing system using a 25 km long large-effective-area fiber (LEAF) sensing fiber coupled with stacked autoencoder-based data processing framework exhibited capabilities in extracting both strain and temperature measurements simultaneously [18]. Further, a denoising autoencoder architecture was shown to both denoise as well as extract temperature from BOTDA data in [11]. Another ANN-based signal processing framework was developed using radial basis functions (RBF) [19,20]. This framework utilizes a shallow neural architecture and requires a much smaller number of parameters compared with deep neural network architectures.

A major drawback of all the existing BOTDA ML models proposed in the literature is that they do not predict the confidence intervals (CI) of the predicted parameters. The reliability of the sensor data and the associated ML model can only be assessed by propagating the measurement noise in the BGS/BPS data to obtain estimates of prediction uncertainties in strain/temperature. Currently, the ability to predict BFS uncertainty from BGS data has only been demonstrated for the simple case of quadratic CF-based BGS processing [21].

We propose a novel *probabilistic machine learning* (PML) framework [22,23,24] for processing BOTDA data, which preserves the existing advantages of ML models while adding a new capability of providing simultaneous CI estimates. The core advantage of the proposed PML framework in this work is the characterization of noise in the data in terms of confidence intervals. The mean and the standard deviation of peak frequency of BFS at each location on the fiber are computed by the PML framework. This enables us to obtain an estimate of the effect of noise in the system at each discrete point on the fiber. First, the mathematical frameworks of the CF and ML approaches are discussed in Section 2, in the context of BFS estimation from BGS and/or BPS measurements. Next, the mathematical framework of the proposed approach is introduced in Section 3. The model development and training procedure are outlined in Section 4. The experimental setup used to generate the data is described in Section 5. The capability of the proposed PML framework to provide simultaneous predictions of BFS and its CI is demonstrated in Section 6 from measurements obtained from two custom BOTDA systems, namely (i) BGS data collected from a 10 km range BOTDA system and (ii) BGS and BPS data collected from a 25 km range custom VBOTDA system. The performance of the PML approach is compared with the CF approach because only these two approaches provide estimates of the parameters and their CIs. Finally, the conclusions and the scope for future work are summarized in Section 7.

## 2. Mathematical Background

Firstly, the mathematical notations adopted in this work are introduced. Let D denote a single BGS and BPS measurement dataset (obtained at a single point along the optical fiber and resolved at *n* frequencies):

(1) $$D\equiv \left\{\left(\nu_{i},g_{i},\phi_{i}\right),\;\forall i=1,\;\ldots \;,n\right\},$$

where frequency, gain, and phase are denoted by $\left(\nu_{i},g_{i},\phi_{i}\right)$, respectively. Let the BFS and Brillouin line width (full width at half maximum (FWHM)) be denoted by $\nu_{B}$ and *w*, respectively. Further, let $\lambda =\left[\lambda_{1}=\nu_{B},\lambda_{2}=w\right]$ denote the vector of parameters of interest. From here on, a single measurement sample of BGS or BPS will be denoted by an input matrix X, wherein

(2) $$X={\left[\begin{array}{ccc} \nu_{1} & \cdots & \nu_{n}\\ g_{1} & \cdots & g_{n} \end{array}\right]}^{⊺},\;or\;\;X={\left[\begin{array}{ccc} \nu_{1} & \cdots & \nu_{n}\\ \phi_{1} & \cdots & \phi_{n} \end{array}\right]}^{⊺},\;or\;\;X={\left[\begin{array}{ccc} \nu_{1} & \cdots & \nu_{n}\\ g_{1} & \cdots & g_{n}\\ \phi_{1} & \cdots & \phi_{n} \end{array}\right]}^{⊺}.$$

**Figure 1:**
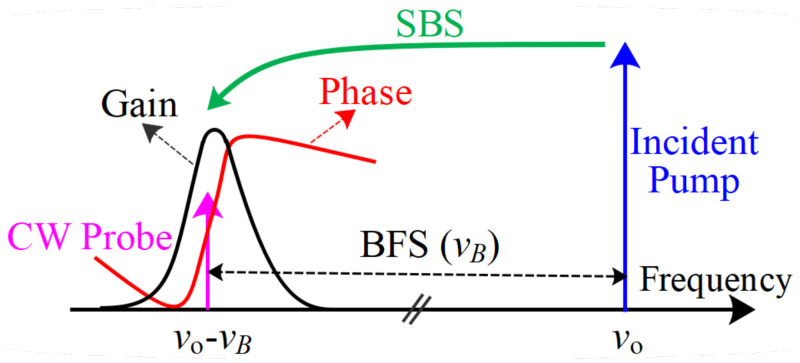
Schematic of Brillouin gain and phase spectrum.

The schematic of the Brillouin gain and phase spectrum is outlined in Figure 1 for illustrative purposes. Next, the mathematical backgrounds of curve fitting and machine learning approaches are briefly discussed below for the case of BGS processing to extract BFS.

### 2.1. Curve Fitting Approach

Curve fitting approaches model the BGS as a smooth parametric function (Lorentzian, Gaussian, and pseudo-Voigt) depending on the parameters of the incident pump light [25,26]. The BGS profile usually has a Lorentzian shape if the pump light pulse width is larger than 10 ns [27], and a Lorentzian fit is often adopted for long-distance BOTDA applications. The BGS profile resembles a Gaussian shape for short incident pulse widths (<10 ns) due to the Doppler broadening effect. The most commonly utilized BGS functions in the literature are given in Table 1.

The BGS measurements are modeled by choosing a suitable BGS function $f_{c}\left(\cdot ;\lambda \right)$ with parameters λ and adding measurement noise as given below:

(3) $$g_{i}=f_{c}\left(\nu_{i};\lambda \right)+\epsilon_{i},$$

where $\epsilon_{i}$ denotes the measurement error. The parameters λ are estimated from the dataset D by using a nonlinear regression based optimization framework:

(4) $$\hat{\lambda }=\underset{\lambda }{\mathrm{argmin}}\frac{1}{n}\sum_{i=1}^{n}{\left(\epsilon_{i}\right)}^{2}=\underset{\lambda }{\mathrm{argmin}}\frac{1}{n}\sum_{i=1}^{n}{\left(g_{i}-f_{c}\left(\nu_{i};\lambda \right)\right)}^{2}.$$

Linear curve fitting using measurements around the zero de-phase frequency region is commonly used to estimate BFS from BPS measurements [9].

The BFS uncertainty from BGS data has only been demonstrated for the simple case of a quadratic BGS profile [21]. However, for other BGS spectra (e.g., Lorentzian, Gaussian etc.), the CIs of the parameter estimates $\hat{\lambda }$ can be estimated using the asymptotic Gaussian distribution of the corresponding least-squares estimator [29]. This asymptotic distribution can be computed from the covariance estimates of the measurement errors ($\epsilon_{i}$ in Equation (3)).

### 2.2. Machine Learning Approach

Machine-learning-based BOTDA data processing algorithms that have been proposed in the literature construct a *direct vectorial functional mapping* $f_{d}\left(\cdot ;\Theta \right)$ between the parameters λ and the BGS dataset X:

(5) $$\lambda =f_{d}\left(X;\Theta \right)+e,$$

where Θ denotes the parameters of the ML model and e denotes the *prediction* error. The training of the ML model requires an annotated dataset containing BGS measurements corresponding to different parameters λ. The ML model parameters are obtained using a suitable optimization framework:

(6) $$\hat{\Theta }=\underset{\Theta }{\mathrm{argmin}}\frac{1}{N}\sum_{i=1}^{N}{∥e_{i}∥}^{2}=\underset{\lambda }{\mathrm{argmin}}\frac{1}{N}\sum_{i=1}^{N}{∥\lambda_{i}-f_{d}\left(X_{i};\Theta \right)∥}^{2},$$

where $\left\{\left(\lambda_{j},X_{j}\right),j=1,\ldots ,N\right\}$ denotes the training dataset of *N* BGS measurements and $∥\cdot ∥$ denotes the Euclidean norm. Upon training, the parameter estimates $\hat{\lambda }$ for a given BGS $\left(X\right)$ can be computed directly using

(7) $$\hat{\lambda }=f_{d}\left(X;\hat{\Theta }\right).$$

Several ML models have been proposed in the literature for the purpose of BFS extraction, including support vector machines [17], principal component regression [30], and artificial neural networks [20,31]. Recent studies in the literature have increasingly advocated for the use of deep neural networks [12,32] with varying architectures such as (i) stacked and denoising autoencoders [11,18], and convolutional neural networks [33,34] to extract BFS from BGS/BPS measurements for low signal-to-noise ratio, long range, and dynamic sensing scenarios.

### 2.3. Comparison of Curve Fitting vs. Machine Learning for BFS Extraction

**Figure 2:**
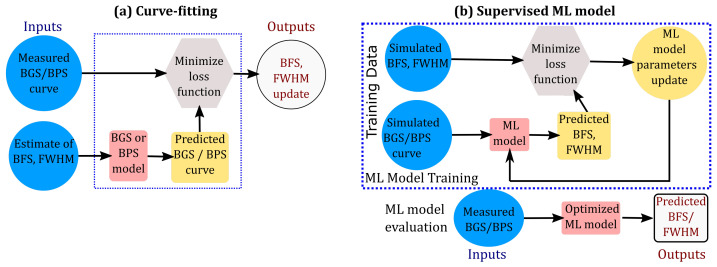
Schematic of (**a**) curve-fitting and (**b**) ML approach to the estimation of BFS and FWHM from BOTDA measurements.

- Data Processing Time: The computational advantage of the ML approach over the CF approach is evident when comparing the schematics of both approaches, as shown in Figure 2. The CF approach requires repeating the optimization iterations for each BGS, while the ML approach can predict the BFS and FWHM directly from the BGS measurements using Equation (7), once the ML model is trained offline.
- Interpretability: However, the CF approach is more interpretable since the function $f_{c}$ can be chosen based on the underlying optical physics knowledge. On the other hand, no such reasoning exists to construct $f_{d}$ in the ML approach and several different ML models have been proposed in the literature.
- Robustness: A key feature of a robust signal processing algorithm is its ability to accurately quantify the confidence/uncertainty in predictions. As mentioned earlier, curve fitting approaches use regression to estimate parameters and can yield CIs of the parameter estimates. However, the ML approach (Equation (5)) can not be used directly to estimate the CIs since the term e does not represent the sensor measurement error. Instead, e in Equation (5) should be interpreted as *prediction error* with an unknown probability distribution due to the nonlinear nature of $f_{d}$. Subsequently, the ML approach provides BFS estimates without providing a measure of uncertainty/confidence (i.e., confidence intervals or error bars) of the BFS predictions. With the increasing adoption of deep neural networks for BOTDA processing, it is even more crucial that the BFS be estimated along with its confidence level, in order to avoid over-fitting.

## 3. Proposed Probabilistic Machine-Learning-Based BFS Extraction

Firstly, the BFS ($\nu_{B}$) and FWHM (*w*) are modeled as statistically independent Gaussian random variables:

(8) $$\lambda \equiv \left[\begin{array}{c} \nu_{B}\\ w \end{array}\right]=\mu_{\lambda }\left(X\right)+\Sigma_{\lambda }\left(X\right)n,\;n=\left[\begin{array}{c} n_{1}\\ n_{2} \end{array}\right]\;n_{1},\;n_{2}∼N\left(0,1\right)$$

where the random variables $n_{1},\;n_{2}$ are independent and identically distributed normal/ Gaussian random variables. The representation in Equation (8) is often referred to as a *reparameterization trick* in the ML literature and is used in several deep learning architectures such as variational autoencoders [35]. The probabilistic model given by Equation (8) provides a pathway to achieve two objectives simultaneously:

1. Mean vector $\mu_{\lambda }$ directly predicts the means of BFS ($\nu_{B}$) and FWHM (*w*) from BGS/BPS measurements X using a suitable ML model
2. Standard deviation matrix $\Sigma_{\lambda }$ quantifies the uncertainty in estimates of BFS ($\nu_{B}$) and FWHM (*w*) due to the noise in underlying measurements X

Subsequently, Equation (8) can be expanded as follows:

(9) $$\mu_{\lambda }\left(X\right)=\left[\begin{array}{c} \mu_{\nu_{B}}\left(X\right)\\ \mu_{w}\left(X\right) \end{array}\right],\;\Sigma_{\lambda }\left(X\right)=\left[\begin{array}{cc} \sigma_{\nu_{B}}\left(X\right) & 0\\ 0 & \sigma_{w}\left(X\right) \end{array}\right]$$

where means of BFS and FWHM are denoted by $\mu_{\lambda }=\left(\mu_{\nu_{B}},\mu_{w}\right)$ and standard deviations of BFS and FWHM are represented in the matrix $\Sigma_{\lambda }$ with diagonal elements $\left(\sigma_{\nu_{B}},\sigma_{w}\right)$. When measurement noise in BGS/BPS data X is minimal, we expect the standard deviations to diminish proportionally $\left(\sigma_{\nu_{B}}\to 0,\;\sigma_{w}\to 0\right)$ and the means to converge to their respective *true* values $\left(\mu_{\nu_{B}}\to \nu_{B},\;\mu_{w}\to w\right)$. In a realistic scenario, wherein there is considerable measurement noise in the Brillouin spectra, we expect the mean values to give the best estimate of the parameter values. At the same time, the standard deviations can be utilized to estimate the level of confidence that could be assigned to the estimated values. Substituting Equation (9) in Equation (8), we obtain

(10) $$\lambda \equiv \left[\begin{array}{c} \nu_{B}\\ w \end{array}\right]=\left[\begin{array}{c} \mu_{\nu_{B}}\left(X\right)+\sigma_{\nu_{B}}\left(X\right)n_{1}\\ \mu_{w}\left(X\right)+\sigma_{w}\left(X\right)n_{2} \end{array}\right].$$

The means and standard deviations (henceforth denoted as Λ) are modeled as functions of BGS and/or BPS measurements, using a suitable ML model:

(11) $$\Lambda \equiv {\left[\begin{array}{cccc} \mu_{\nu_{B}} & \mu_{w} & \sigma_{\nu_{B}} & \sigma_{w} \end{array}\right]}^{⊺}=f_{d}\left(X;\Theta \right).$$

wherein the parameters of the ML model are denoted by Θ. Using Equation (11) in Equation (10), it can be realized that the parameters of interest λ are represented using a *probabilistic machine learning* (PML) model.

**Figure 3:**
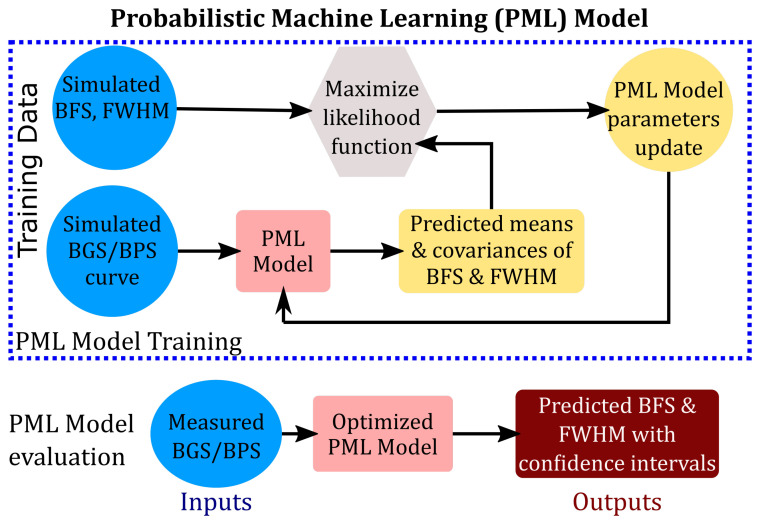
Schematic of PML approach to the estimation of BFS and FWHM with confidence intervals from BOTDA measurements.

The proposed procedure to estimate the PML model parameters Θ is depicted in Figure 3. We utilize an offline training framework to train the PML model. A synthetic/simulated training dataset is obtained by using Lorentzian BGS and/or BPS curves for various BFS, FWHM parameters, and noise levels. Let $\left\{\left(\lambda_{j},X_{j}\right),j=1,\ldots ,N\right\}$ denotes the training dataset of *N* BGS/BPS measurements corresponding to various values of BFS, FWHM, and noise values. It should be noted that each $X_{j}$ is a matrix of *n* frequencies and corresponding gain and/or phase values, as given in Equation (2). The PML parameters cannot be estimated using deterministic loss functions as employed by CF (Equation (4)) and ML approaches (Equation (6)). This work proposes the use of the probabilistic objective function known as the log-likelihood function:

(12) $$\hat{\Theta }=\underset{\Theta }{\mathrm{argmax}}\frac{1}{N}\sum_{i=1}^{N}\log\left[p\left(\lambda_{i};\Lambda =f_{d}\left(X_{i};\Theta \right)\right)\right]$$

where $p\left(\lambda_{i}|\Lambda \right)$ denotes the joint probability density function of BFS and FWHM for a given set of parameters Λ. From Equation (11), we can write the joint Gaussian probability density function as follows:

(13) $$p\left(\lambda \right)\equiv p\left(\nu_{B},w\right)=\frac{1}{2\pi }\det{\left(\Sigma_{\lambda }\right)}^{-0.5}\exp\left(-\frac{1}{2}{\left(\lambda -\mu_{\lambda }\right)}^{⊺}\Sigma_{\lambda }^{-1}\left(\lambda -\mu_{\lambda }\right)\right).$$

By maximizing the log-likelihood function, we obtain a joint probability distribution that maximizes the probability of $\lambda_{i}$ for a given $X_{i}$. Using Equation (13) in Equation (12), we obtain a least-squares objective function:

(14) $$\hat{\Theta }=\underset{\Theta }{\mathrm{argmax}}\frac{-1}{2N}\sum_{i=1}^{N}\left[2\log\left(2\pi \right)+\log\sigma_{\nu_{B}}+\log\sigma_{w}+{\left(\frac{\nu_{B_{i}}-\mu_{\nu_{B}}}{\sigma_{\nu_{B}}}\right)}^{2}+{\left(\frac{w_{i}-\mu_{w}}{\sigma_{w}}\right)}^{2}\right]$$

Upon training by maximizing Equation (14), the estimates of the means and standard deviations of BFS and FWHM can be computed by evaluating the PML model as depicted in Figure 3:

(15) $$\hat{\Lambda }\equiv {\left[\begin{array}{cccc} {\hat{\mu }}_{\nu_{B}} & {\hat{\mu }}_{w} & {\hat{\sigma }}_{\nu_{B}} & {\hat{\sigma }}_{w} \end{array}\right]}^{⊺}=f_{d}\left(X;\hat{\Theta }\right).$$

The point estimates and 100(1−α)% CI estimates of BFS and FWHM can be computed from Equation (15) as follows:

(16) $$\begin{array}{cc} & \hat{\lambda }={\left[\begin{array}{cc} {\hat{\mu }}_{\nu_{B}} & {\hat{\mu }}_{w} \end{array}\right]}^{⊺} \end{array}$$

(17) $$\begin{array}{cc} & {\hat{\mu }}_{\nu_{B}}-s_{\alpha }{\hat{\sigma }}_{\nu_{B}}\leq \nu_{B}\leq {\hat{\mu }}_{\nu_{B}}+s_{\alpha }{\hat{\sigma }}_{\nu_{B}} \end{array}$$

(18) $$\begin{array}{cc} & {\hat{\mu }}_{w}-s_{\alpha }{\hat{\sigma }}_{w}\leq w\leq {\hat{\mu }}_{w}+s_{\alpha }{\hat{\sigma }}_{w} \end{array}$$

where $s_{\alpha }=\Phi \left(1-\frac{\alpha }{2}\right)$ is the statistic computed from a standard normal cumulative distribution function Φ.

This probabilistic framework has computational benefits in comparison with other PML approaches [22] such as dropout [36], bootstrapping [37] and weight randomization [38]. The PML approach has several advantages over CF and ML approaches, as listed below:

1. **Robustness**: The PML approach prevents overfitting that arises when using ML and DNN models to represent $f_{d}$.
2. **Flexibility**: It is compatible with the various ML models (e.g., neural networks [39] and support vector regression [40]).
3. **Speed**: It inherits the computational advantages of the ML approach and enables fast processing of BOTDA data with simultaneous assessment of prediction uncertainties.

## 4. PML Model Development and Training

A deep neural network (DNN) with a combination of convolutional and dense layers is chosen to represent $f_{d}$ in Equation (11). It is evident from Equations (10) and (11) that the outputs of the DNN are the means and standard deviations of BFS and FWHM. Consequently, the DNN will henceforth be known as a *probabilistic deep neural network (PDNN)*. The PDNN was programmed in Python and the code is available in Code 1 [41]. This PML framework can be easily implemented with another ML or DNN model with minor modifications in the code.

### 4.1. PML Model Training

This work utilizes a simulated dataset of BGS and BPS measurements corresponding to a uniform grid of BFS, FWHM, and noise values. All of the parameters are sampled from uniform probability distributions $\left(y∼U\left(y_{l},\;y_{u}\right),\;p\left(y\right)=\frac{1}{y_{u}-y_{l}},\;\forall y_{l}\leq y\leq y_{u}\right)$ between lower and upper bounds, as given below:

(19) $$s∼U\left(\frac{1}{18},\frac{1}{5}\right),\;\nu_{B}∼U\left(10,50\right)MHz,\;w∼U\left(5,50\right)MHz$$

It should be noted that in addition to varying the BFS and FWHM values in a uniform grid, we also vary noise amplitude levels. The variation of BFS and FWHM is essential to predict the mean vector $\mu_{\lambda }$, and the variation of noise amplitude is essential to predict $\Sigma_{\lambda }$.

We generate the training dataset by first choosing a vector of frequency values to compute BGS and/or BPS: $\nu_{1},\ldots ,\nu_{K}$ and training sample size *N*. We have considered a large training sample size of N=50,000.

For j=1…N:

1. Uniformly sample *s*, $\nu_{B}$, and *w* from the bounds in Equation (19) to obtain $\left(s_{j},{\nu_{B}}_{j},w_{j}\right)$
2. Simulate gain and phase values for each of the *n* frequencies and for $\left({\nu_{B}}_{j},w_{j}\right)$ using a suitable spectrum model.
  (20) $$\begin{array}{cc} g_{j}\left(\nu_{i}\right) & =g\left(\nu_{i};{\nu_{B}}_{j},w_{j}\right),\;\forall i=1\ldots K \end{array}$$
  (21) $$\begin{array}{cc} \phi_{j}\left(\nu_{i}\right) & =\phi \left(\nu_{i};{\nu_{B}}_{j},w_{j}\right),\;\forall i=1\ldots K \end{array}$$
  This work has chosen Lorentzian BGS and BPS [7] (Equations (22) and (23)) given by the following:
  (22) $$\begin{array}{cc} g\left(\nu ;\nu_{B},w\right) & =g_{0}\frac{w^{2}}{4{\left(\nu -\nu_{B}\right)}^{2}+w^{2}} \end{array}$$
  (23) $$\begin{array}{cc} \phi \left(\nu ;\nu_{B},w\right) & =g_{0}\frac{2w\left(\nu -\nu_{B}\right)}{4{\left(\nu -\nu_{B}\right)}^{2}+w^{2}} \end{array}$$
  where $g_{0}$ is the Brillouin gain amplitude, *w* is the Brillouin linewidth or FWHM, and $\nu_{B}$ is the BFS.
3. Sample $e∼N\left(0,1\right)$ and add Gaussian noise corresponding to the noise amplitude $s_{j}$ to obtain training dataset sample $X_{j}$
  (24) $$\begin{array}{c} X_{j}={\left[\begin{array}{ccc} \nu_{1} & \cdots & \nu_{K}\\ g_{j}\left(\nu_{1}\right)+s_{j}e & \cdots & g_{j}\left(\nu_{K}\right)+s_{j}e\\ \phi_{j}\left(\nu_{1}\right)+s_{j}e & \cdots & \phi_{j}\left(\nu_{K}\right)+s_{j}e \end{array}\right]}^{⊺} \end{array}$$

### 4.2. PML Model Architecture

**Figure 4:**
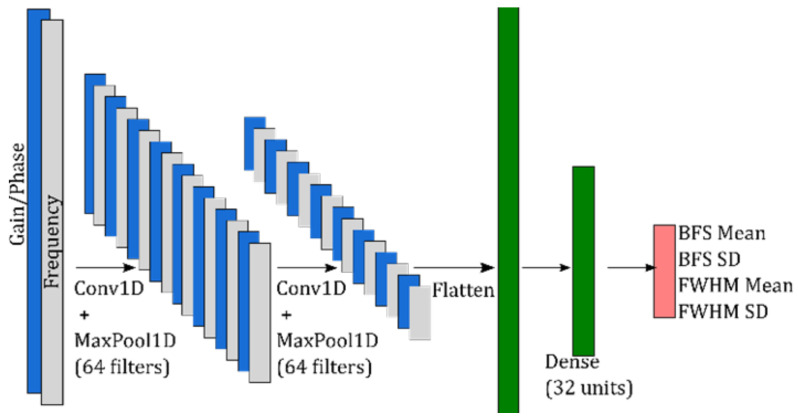
PML model architecture.

The PDNN architecture is depicted in Figure 4. It has a combination of convolutional and dense layers. The input layer consists of two channels. The first 1D convolutional layer (Conv1D) has a kernel size of 15 and (Rectified Linear Unit) ReLU activation. The second Conv1D has a kernel size of 5, and both MaxPool1D layers have kernel size = 3. The dense layer has 32 units, with ReLU and $L_{2}$ regularization.

The PDNN is trained over this dataset using a training/validation split of 70/30. Further, a learning rate schedule and early stopping criterion are implemented to automate the model training.

## 5. Experimental Setup

**Figure 5:**
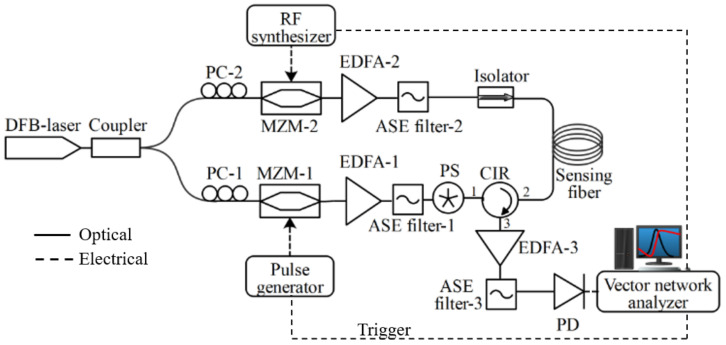
Experimental setup of VBOTDA system. DFB-laser: distributed feedback-laser, PC: polarization controller, MZM: Mach–Zehnder modulator, EDFA: Erbium-doped fiber amplifier, ASE: amplified spontaneous emission, PS: polarization scrambler, CIR: circulator, PD: photo-detector).

The experimental setup of the vector Brillouin optical time domain analysis (VBOTDA) system is illustrated in Figure 5. In [10], we demonstrated a VBOTDA system, where a vector network analyzer was used to extract both the amplitude and phase spectrum of the Brillouin interaction over the sensing fiber.

A distributed feedback (DFB) laser with a wavelength of 1550 nm was used as a laser source. The laser output signal was split into two branches using a 50/50 3 dB fiber coupler. One branch was for generating input pump pulses and the other branch was for the probe signal to make stimulated Brillouin operation over the fiber distance. A Mach–Zehnder modulator (MZM-1) was used to modulate the optical pulses using a pulse generator. The pulse peak-to-peak amplitude set at 4 Vpp, and the pulse width was set at 10 ns, corresponding to 1 m spatial resolution, whereas the pulse repetition frequency was 4 kHz, which has enough round-trip time to travel the full length of the fiber. In order to compensate the high lossy operation of MZM modulators, a polarization controller (PC) was employed at the input of each MZM to alter the polarization state of the signal and to reach maximum optical power after both MZMs. The output signal was then amplified using an erbium-doped fiber amplifier (EDFA-1). A narrow band-pass filter bandwidth: 0.8 nm was employed to reduce the amplified spontaneous emission (ASE) noise originating from the EDFA. To avoid polarization noise due to the intense polarization sensitivity of the stimulated Brillouin scattering mechanism, a high-speed polarization scrambler (PS) was used to reduce the polarization dependence of Brillouin spectra. The second MZM (MZM-2) was modulated with a frequency close to the BFS of the sensing fiber (10.82 GHz) via an external RF synthesizer. The signal was amplified by EDFA-2 and then sent to an isolator, which allows signal transmission in one direction. The input pump peak power (20 dBm) and the counter-propagating probe power (6 dBm) were sent to the fiber under test. The stimulated Brillouin signal from the circulator (CIR) port 3 was amplified using EDFA-3 and sent to ASE filter-3 to remove the ASE noise. Thereafter, the signal was detected using a photodetector (PD, bandwidth: 125 MHz) and analyzed with a vector network analyzer. In order to obtain the BGS spectra over the fiber distance, the RF synthesizer frequencies were swept around fiber under test BFS, which were 10.78 GHz to 10.9 GHz with a frequency step of 1 MHz. The RF synthesizer and pulse generator were synchronized with the vector network analyzer. For the fiber under test, we used two different lengths of sensing fibers, 10 km and 25 km, and obtained 3D BGS specta over the sensing fiber distance at various trace averages. The sensing fiber was kept at room temperature and under strain-free condition throughout all the measurements.

## 6. Results and Discussions

The PML framework is illustrated to process data using (i) a custom BOTDA system (BGS data) with a 10 km long sensing fiber with trace averages of 10 and 100 independently [42] and (ii) a custom VBOTDA system (BGS, and BPS data) with a 25 km range with 1000 trace averages. The results for both cases are presented and analyzed below:

### 6.1. Custom BOTDA System Using 10 km Long Sensing Fiber

**Figure 6:**
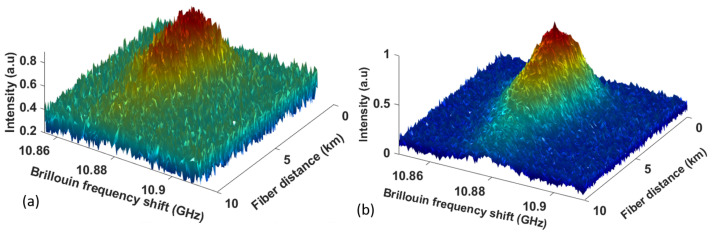
Measured 3D BGS spectra using (**a**) 10 trace averages and (**b**) 100 trace averages.

**Figure 7:**
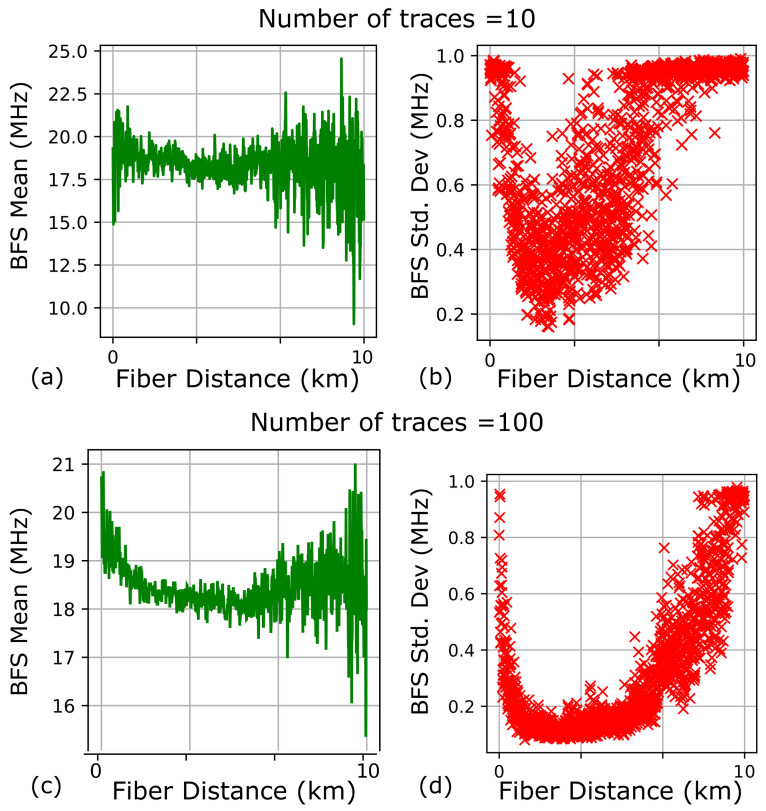
PDNN estimates of mean (**a**,**c**) and standard deviations (Std. Dev) (**b**,**d**) from two 10 km BOTDA datasets. (**a**,**b**) PDNN estimates from BGS data averaged over 10 traces. (**c**,**d**) PDNN estimates from BGS data averaged over 100 traces.

The three-dimensional BGS was constructed with a sweep frequency step of 1 MHz with different trace averages of 10 and 100. The resultant BGS spectra are illustrated in Figure 6. The mean ${\hat{\mu }}_{\nu_{B}}$ and standard deviations ${\hat{\sigma }}_{\nu_{B}}$ of the BFS along the 10 km fiber length obtained from the two datasets are plotted in Figure 7. The two datasets are obtained for various trace averages (10 traces for Figure 7a,b and 100 traces for Figure 7c,d). It should be noted that the first few tens of meters have high standard deviations due to the dead zones [43] in Figure 7. As the fiber distance increases, the standard deviations escalate due to fiber attenuation, resulting in diminishing signal-to-noise ratio. It is also clear from Figure 7b,d that the PDNN’s BFS uncertainty estimates reflect the well-documented phenomenon [9] that BFS uncertainty reduces with an increase in the number of traces. The proposed approach with confidence interval outputs at every location on the fiber quantitatively estimates the performance metrics of the fiber optic sensor. This information can be used to mitigate any defects and to enhance the performance of the fiber optic sensor along the length of the pipeline.

### 6.2. Custom VBOTDA System Using 25 km Long Sensing Fiber

**Figure 8:**
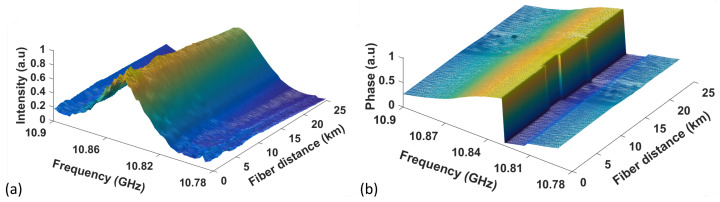
Measured 3D (**a**) BGS and (**b**) BPS over 25 km fiber.

The BGS and BPS obtained over the fiber length are available as Dataset 1 [44] and Dataset 2 [45] respectively. Both are plotted in Figure 8.

**Figure 9:**
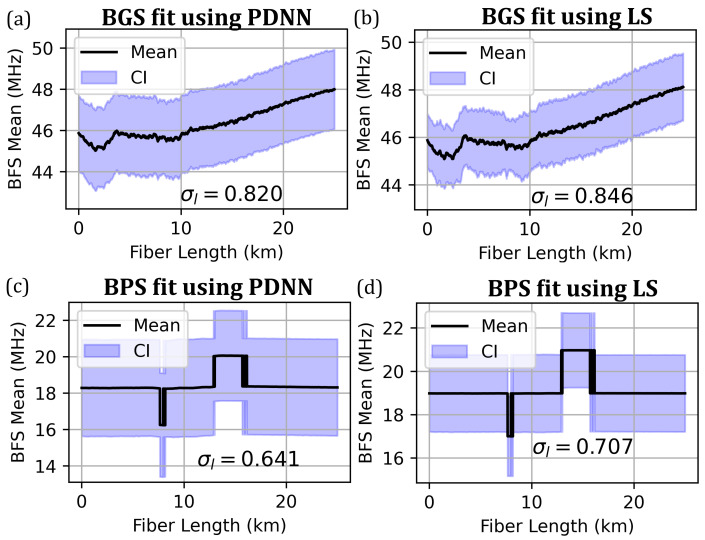
Comparison of BFS extracted using PDNN (**a**,**c**) and LS fit (**b**,**d**) from BGS or BPS measurements. The unit of confidence interval $\sigma_{l}$ is MHz.

**Figure 10:**
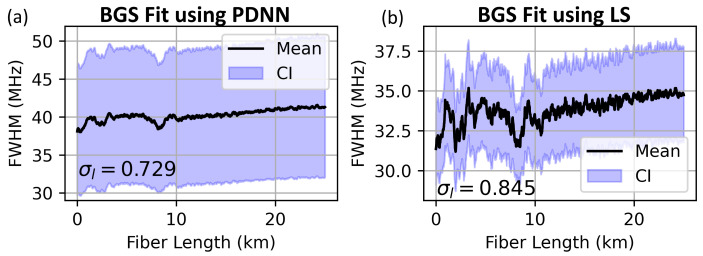
FWHM predictions using (**a**) PDNN and (**b**) LS fit from BGS data. The unit of confidence interval $\sigma_{l}$ is MHz.

The mean BFS (computed with respect to 10.8 GHz) is predicted using the PDNN model over the entire fiber length from the BGS and BPS spectra and is plotted with $\pm 3\sigma_{\nu_{B}}$ (99.7%) confidence intervals in Figure 9a,c. These predictions are compared with a least-squares (LS) Lorentzian curve fitting model, as shown in Figure 9b,d. FWHM predictions from both models are shown in Figure 10.

The mean BFS predictions from PDNN (for BGS and BPS) are in excellent agreement with those from LS fits. The PDNN processes 4500 spectra in 1.1 s (for both BGS and BPS), while the CF approach takes 13.9 s and 18.5 s, respectively. The total computational speedup achieved with the PDNN approach is around 30 times more compared with the curve fitting approach. This quantum of speedup enables us to process the data in real time in the field as we collect sensor data all along the pipeline. Clearly, the PDNN model greatly reduces the data processing time. Furthermore, the BPS-based predictions exhibit lesser spatial uncertainty compared with that of BGS-based predictions.

## 7. Conclusions

We proposed and demonstrated a novel robust signal processing framework using PML to estimate the BFS from BOTDA systems. The PML is capable of processing BGS and BPS spectra in real time. Further, unlike ML models, which do not propagate uncertainties, our proposed PML model can provide estimates of predictive uncertainties. We compared the predictions obtained from the proposed PML with the conventional CF model and evaluated the BFS uncertainty and data processing time for both methods. The proposed PML model offers greater tolerance to measurement noise found in real-time strain and temperature extraction for a longer sensing range. Hence, the PML framework can be used to build a robust signal processing system and provides a pathway to enhance the VBOTDA system performance. Future advancements in ML techniques integrated with BOTDA sensor technology will continue to drive the evolution of distributed fiber optic sensors, enabling even more sophisticated and intelligent sensing capabilities. We plan to incorporate efficient data denoising techniques within the proposed PML framework in the future.

## Figures and Tables

**Table 1:** Commonly used BGS [28].

Gain Spectrum	Function	Parameters
	$f_{c}\left(\nu ;\lambda \right)$	λ
Lorentzian	$f_{c}\left(\nu ;\lambda \right)=\frac{g_{0}}{1+4\xi^{2}},\;\xi =\frac{\left(\nu -\nu_{B}\right)}{w}$	$\lambda \equiv \left[g_{0},\nu_{B},w\right]$
Gaussian	$f_{c}\left(\nu ;\lambda \right)=g_{0}\exp\left[-4\ln2\xi^{2}\right],\;\xi =\frac{\left(\nu -\nu_{B}\right)}{w}$	$\lambda \equiv \left[g_{0},\nu_{B},w\right]$
Pseudo-Voigt	$f_{c}\left(\nu ;\lambda \right)=g_{0}\left[\frac{p}{1+4\xi^{2}}+\left(1-p\right)\exp\left(-4\ln2\xi^{2}\right)\right]$	$\lambda \equiv \left[p,g_{0},\nu_{B},w\right]$

## Data Availability

Data presented in this paper are available as Datasets 1–2 [44,45] and the code is available in Code 1 [41].
